# Laterality of blood perfusion in the lower extremities after drinking saline at different temperatures

**DOI:** 10.1038/s41598-023-28758-y

**Published:** 2023-01-28

**Authors:** Shuyong Jia, Qizhen Wang, Hongyan Li, Xiaojing Song, Shuyou Wang, Weibo Zhang, Guangjun Wang

**Affiliations:** 1 Institute of Acupuncture and Moxibustion, China Academy of Chinese Medical Sciences, Beijing, China; 2 Institute of Basic Research in Clinical Medicine, China Academy of Chinese Medical Sciences, Beijing, China

**Keywords:** Preclinical research, Translational research

## Abstract

Skin blood flux (SkBF) changes caused by drinking cold water are generally associated with vagal tone and osmotic factors in the digestive system. However, there is still a lack of relevant research on whether there are left and right differences in these SkBF change. In the current study, a total of 60 subjects were recruited. Skin blood perfusion of the bilateral lower extremities was recorded simultaneously before and after drinking saline of different temperatures saline by using Laser Doppler flowmetry (LDF). The electrogastrogram (EGG) was also monitored, and the dominant frequency of the EGG and heart rate variability were analyzed. The results indicated that after drinking saline, the laterality index of SkBF at the lower extremities was different and the laterality index changes of SkBF were mainly reflected in the frequency interval V (0.4–1.6 Hz). There was a weak negative correlation between the laterality index of endothelial NO-dependent component and change rate of root mean square of successive differences (RMSSD) after drinking 4 °C saline. However, after drinking 30 °C saline, there was a weak positive correlation between neurogenic component and RMSSD The distribution and regulation of bilateral blood flow are not symmetrical but exhibit a certain laterality.

## Introduction

Although people prefer to drink cold water during endurance exercise^1^, few reports have studied the effects of drinking water of temperatures in healthy humans^2–4^. Studies about the effect of water temperature on blood flux are especially scarce. Scientists have suggested that the water effects, such as decreased heart rate, increased total peripheral resistance and baroreceptor sensitivity, do not depend on gastric distension, but on water osmolality, which may cause an autonomic cardiovascular response in humans through osmotic sensing nerve fibers in the intestinal or portal circulation^5^. On the other hand, results have shown that water intake activates distinct gastrointestinal vagal afferents in a temperature-dependent pattern and might affect cardiac vagal tone^6^.

One previous report suggested that oral glucose intake inhibits hypothalamic neuronal activity more effectively than intravenous (IV) glucose administration^7^, but compared to IV infusion, oral intake of saline results in minimal variations in serum albumin, hematocrit, and hemoglobin^8^. These studies indicated that, in addition to osmotic pressure and temperature mechanisms, the different autonomic responses of the digestive tract might result in systemic regulation of peripheral blood perfusion. Therefore, skin blood flow can be used to explore the regulation of gastrointestinal autonomic function after stimulation. According to previous studies^9,10^, the cutaneous blood perfusion is different on the right and left side of the body.

The activation of the sympathetic or parasympathetic nervous system has a direct effect on gastrointestinal motility^11,12^, and the automatic nervous system significantly affects electrogastrogram (EGG) frequency^13^. One previous study that water ingestion might modulate gastric motility^14^ and EGG frequency^15,16^. Cold stimulation of the digestive tract may not only cause a change in the EGG, but also cause systemic reactions. However, it is still unclear whether EGG power is well related to automatic nervous system function under cold water ingestion^17^.

It has been found that a large number of vagal afferent fibers are distributed in the proximal gastrointestinal tract^18^, and some fibers are sensitive to temperature stimulation^19,20^. Ice water ingestion can induce cardioautonomic responses^21^ and low temperature water drinking can decrease heart rate through the vagal nervous system^22^. As part of the cardioautonomic response, skin blood flow regulation^23^ and heart rate are all regulated by the autonomic nervous system. Due to the different adaptability of blood flow on different sides of the body due to changes in position^24^, we hypothesized that as another environmental change, temperature stimulation in the digestive tract can also induce different responses of blood perfusion on different sides, showing a certain laterality. To test these hypotheses, we conducted a randomized control study to compare the cutaneous blood perfusion changes in response to saline of different temperatures and we also evaluated the relationship between EGG dominant frequency (DF) and cardiovascular variables.

## Methods

### Ethics approval

This study was approved by the Institutional Research Ethics Boards of Acupuncture & Moxibustion, China Academy of Chinese Medical Sciences. In accordance with the Declaration of Helsinki^25^, each subject provided informed consent and had an adequate understanding of the procedure and purpose of this study.

### Inclusion and exclusion criteria for subjects

The subject cannot be pregnant, and the measurement cannot be carried out during menstruation The inclusion criteria were good health and an age between 18 and 60 years. The exclusion criteria were presence of diseases affecting cardiovascular or autonomic regulation, administration of any medication affecting cardiovascular or autonomic regulation, pregnancy, and menstruation at time of testing^26^.

### Participants and design

**Figure 1:**
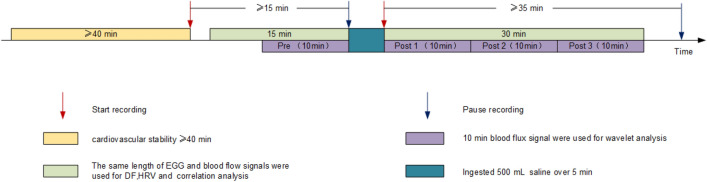
Experiment design and process of signal recording and analysis. EGG, electrogastrogram; DF, dominant frequency; HRV, heart rate variability.

A total of 60 healthy subjects were enrolled in the study, and all subjects completed the measurements and were included in the statistical analysis. All subjects lived in Beijing, and all were Chinese. The general characteristics are presented in Table 1. All experiments took place in a quiet, temperature-controlled (24–26 °C) laboratory. On arrival at the laboratory, subjects were asked to empty their bladders. Following a period of cardiovascular stability (40 min), a baseline recording was undertaken for at least 15 min. Then, over a 5–min period, the test subjects of different groups ingested 500 mL of 4 °C, 10 °C, or 30 °C 0.9% saline, respectively, and EGG and SkBF were monitored at least for 35 min. The experimental design, signal recording and analysis process are shown in Fig. 1. In order to analyze the dynamic changes in SkBF after stimulation, 10-min recordings were taken as units of the analysis and named Pre, Post 1, Post 2 and Post 3, respectively. In addition, data were not segmented in the EGG analysis, heart rate variability (HRV) analysis, and the correlation analysis with SkBF, and 15-min baseline and 30-min post-stimulation recordings were used for analysis.

**Table 1:** Distribution of sex, age, height and weight.

Group (°C)	n	Sex (female/male)	Age (years, mean ± SD)	Height (cm, mean ± SD)	Weight (kg, mean ± SD)
4	20	17/3	27 ± 3	165.0 ± 7.2	59.6 ± 9.3
10	20	18/2	25 ± 3	163.6 ± 5.6	56.2 ± 10.7
30	20	17/3	26 ± 1	161.9 ± 6.1	54.6 ± 13.6

### Electrogastrogram measurement protocol and analysis

All subjects were advised to avoid alcohol, tea, and coffee for at least 24 h prior to the test. The subjects maintained a supine position for measurements. Eight cutaneous electrodes were placed on the abdomen of the subject and connected to an amplifier (EXG model, MEGA Electronics Ltd, Kuopio, Finland) with direct-current mode. The EGG recordings were processed with a NeurOne system (NeurOne, MEGA Electronics Ltd, Finland). The data were digitized with a sampling rate of 10,000 Hz and then down-sampled at 1000 Hz. EGG data were analyzed using the FieldTrip toolbox^27^, and the analysis code for EGG analysis was provided by Wolpert et al. (https://github.com/niwolpert/EGG_Scripts)^28^. For each channel, a fast Fourier transform (FFT) algorithm was implemented in the Fieldtrip toolbox with a Hanning taper to estimate the spectral power of EGG. The largest activity in the 0.033–0.066 Hz (2–4 cpm) was used to determine the dominant frequency (DF) of each channel. FFT was carried out on all channels, and eight wave peaks were obtained. The frequency corresponding to the maximum wave peak value represented the DF of each subject.

### Root mean square of successive differences (RMSSD) and mean RR interval analysis

The analysis method can be referenced in previous study^29^. The raw data of 1 channel was exported in European data format (EDF) format, imported into Kubio HRV software (Kubios Oy, Kuopio, Finland) and analyzed^30–32^. The data were detrended using the smoothness prior approach with a lambda value of 500 and artifacts were corrected by applying the medium filter provided by Kubios HRV. RMSSDwas calculated by (1). Both the mean RR and RMSSD change rates were calculated by (2).

1 $$RMSSD = \sqrt {\frac{1}{N - 1}\sum\limits_{n = 1}^{N - 1} {(RR_{n + 1} - RR_{n} )^{2} } }$$

2 $$ChangeRate = \frac{Post}{{Pre}} \times 100$$

### Protocol for measurement of blood perfusion

For measurements, both legs were exposed, and bilateral Zusanli acupoints (ST 36), which are located in the tibialis anterior muscle, 4 fingerbreadths below the kneecap and 1 fingerbreadth lateral from the anterior crest of the tibia^33^, were marked by senior acupuncture doctors. From the perspective of traditional acupuncture theory, ST 36 is closely related to the digestive system. However, in the current study, the aim was not related to traditional acupuncture theory, so we do not discuss the specificity of ST 36, and it was simply used as an observation point of the low extremities. Blood perfusion signals were recorded using a PeriFlux System 5000 (Perimed AB, Stockholm, Sweden) with a 64 Hz sample rate and 0.2 s time constant. An optical fiber probe connected with a Periflux 5000 was used to illuminate and collect the scattered light from the skin tissue. The probe was attached to the surface of interest with two-sided adhesive tape.

### Mean blood perfusion analysis and wavelet analysis

The recorded file of each subject was opened in PeriSoft (version 2.5.5, Perimed AB, Stockholm, Sweden) for Windows. The detailed data were exported in txt format and then imported into MATLAB software and analyzed. The laterality index of blood flux every 10 min on both sides of ST36 was calculated by (3).

3 $$Laterality\, Index = \frac{{mean(\left| {Post_{L} - Post_{R} } \right|)}}{{mean(\left| {Pre_{L} - Pre_{R} } \right|)}} \times 100$$

Previous studies have indicated that blood flux oscillations at frequencies from 0.0095 to 1.6 Hz can be separated into six frequency bands in the frequency domain^34^. In the present study, wavelet analysis was performed on the blood flux signal (10 min) using a Morlet wavelet (MathWorks, Natick, MA, USA). The component values corresponding to different frequencies were obtained after wavelet transform and frequency domain averaging For every frequency interval, the laterality index was also calculated as (3).

### Statistical analysis

Data are presented as the mean ± SE. A paired t-test was used to comparepre- and post-stimulation. Mixed repeated-measures analysis of variance (ANOVA) was used to analyze between-subject factors with R software^35^. The normality of data was judged by Shapiro-Wilks test in R package. Statistical power was analyzed using G*Power 3.1 software^36^. The correlation between the laterality index and RMSSD or DF was analyzed using Spearman’s rank correlation coefficient. All correlation analyses were conducted using MATLAB software. All reported *P* values were two-sided and were corrected using the FDR method using fdrtool package^37^. The level of significance was defined as *P* < 0.05.

## Results

In this study, a total of 60 subjects were recruited, and all subjects were included in the final statistical analysis. Detailed information on the subjects is summarized in Table 1 (4 °C, N = 20; 10 °C, N = 20 and 30 °C, N = 20).

### EGG and ECG results

**Figure 2:**
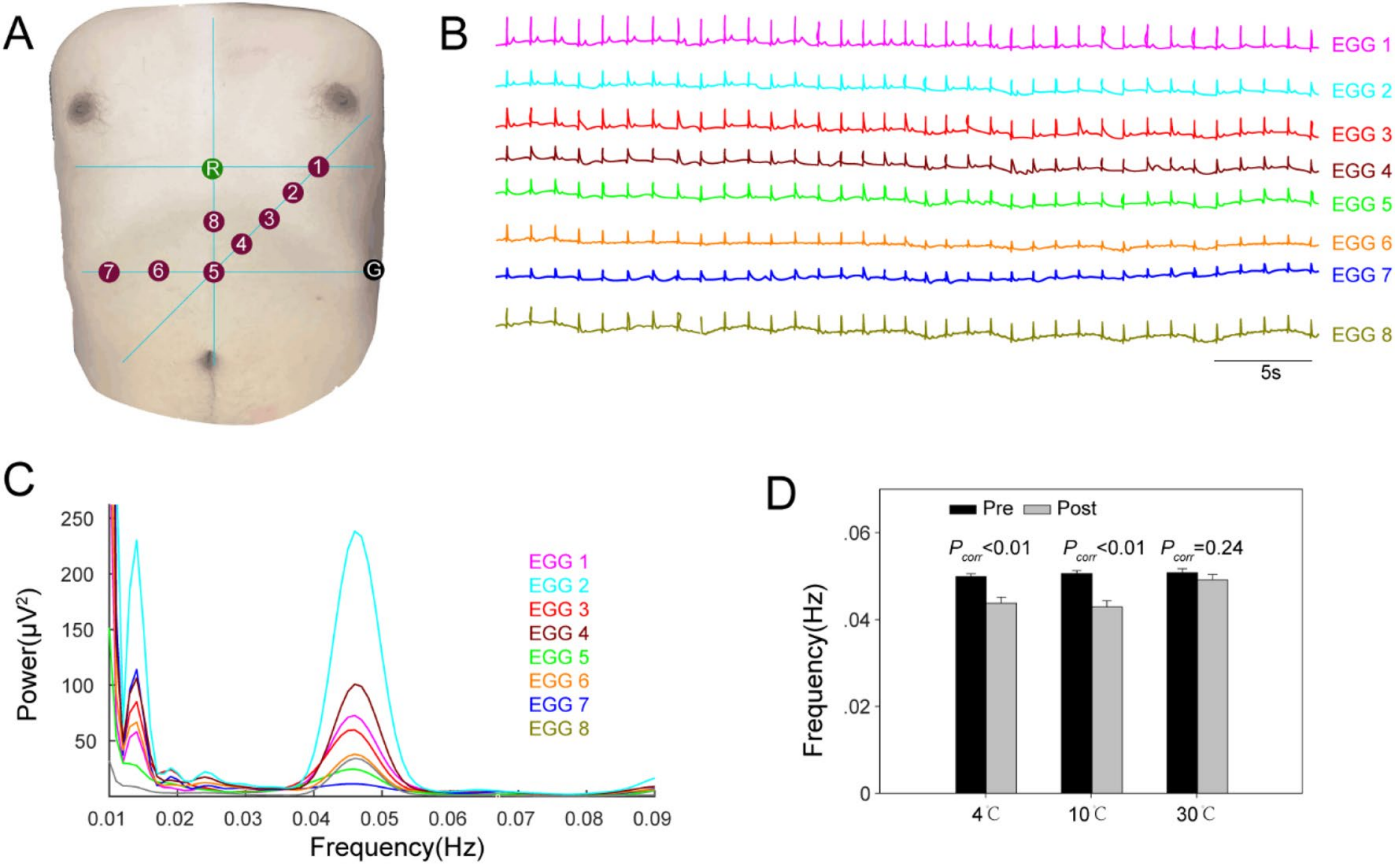
EGG power spectra changes. (**A**) Cutaneous electrode position; (**B**) raw data of EGG; (**C**) power spectra of EGG; (**D**) EGG peak frequency before and after stimulation. *P*_*corr*_ < 0.01 (4 °C); *P*_*corr*_ < 0.01 (10 °C); *P*_*corr*_ = 0.24 (30 °C); *P*_*corr*_: corrected *P* value. EGG, electrogastrogram.

The results of the EGG analysis are shown in Fig. 2, the recording electrode position is shown in Fig. 2A, and the raw data of the 8-channel gastrointestinal electrical signals recordings are shown in Fig. 2B. The spectral analysis of each signal is shown in Fig. 2C. The statistical results of the main frequency corresponding to the maximum power in each subject's 8-channel gastrointestinal electrical signals are shown in Fig. 2D. In the resting state, there was no significant difference in the DF of the subjects.

**Figure 3:**
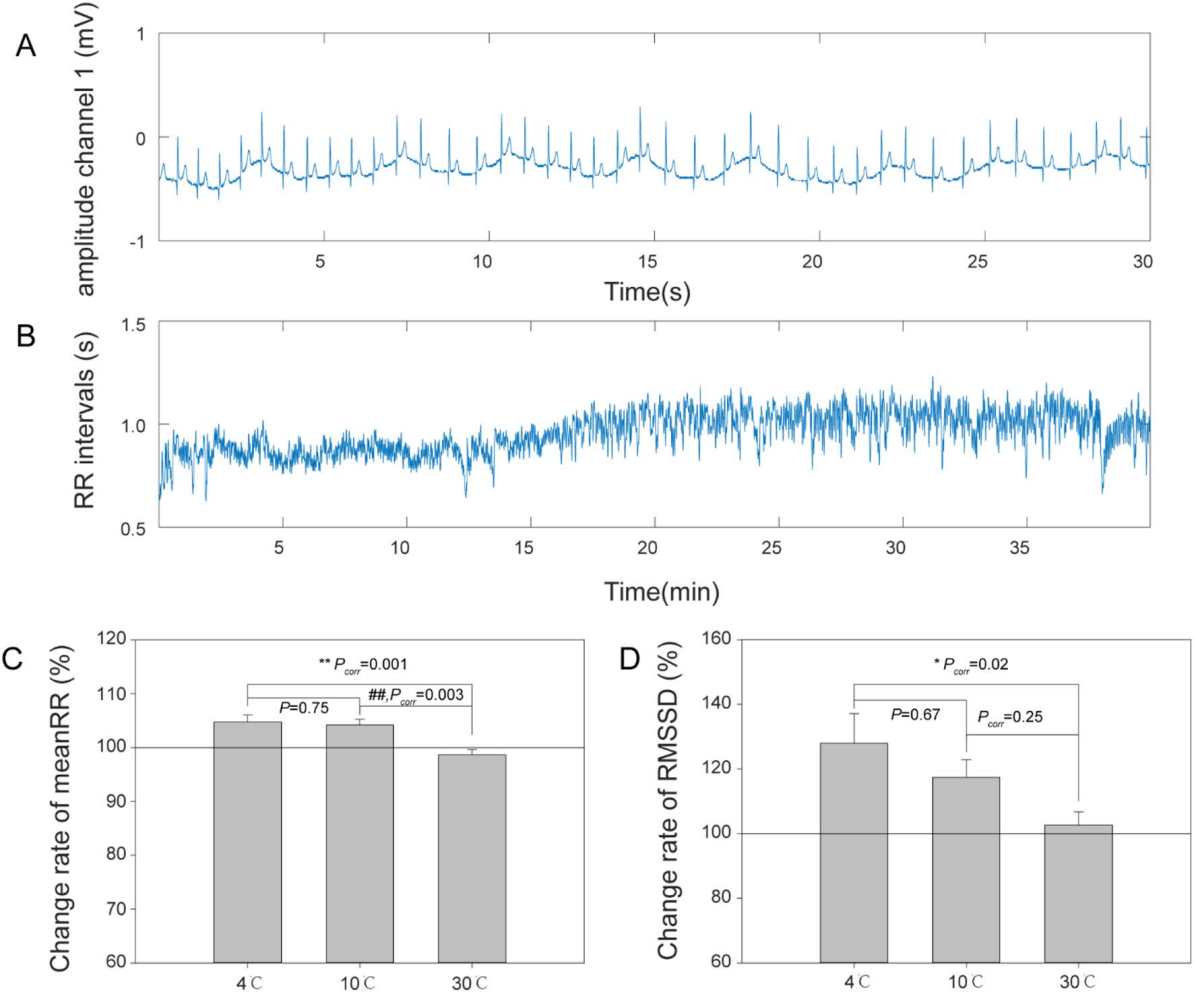
Change in HRV. (**A**) ECG signal from channel 1; (**B**) RR intervals of ECG; (**C**) Change rate of mean RR, *F*_(2,57)_ = 8.73, *P* = 0.0005; **P_*corr*_ < 0.01; ##*P*_*corr*_ < 0.01; (**D**) Change rate of RMSSD; *F*_(2,57)_ = 3.75, *P* = 0.029; *P_*corr*_ < 0.05; *P*_*corr*_: corrected *P* value. All values are reported as the mean ± standard error. ECG, electrocardiogram; RMSSD, root mean square of successive differences; HRV, heart rate variability.

The raw ECG and RR interval signal data are shown in Fig. 3A,B, respectively. Compared with 30 °C stimulation, the RR interval (Fig. 3C) and RMSSD (Fig. 3D) were increased with both 4 °C and 10 °C stimulation.

### Skin blood flux

**Figure 4:**
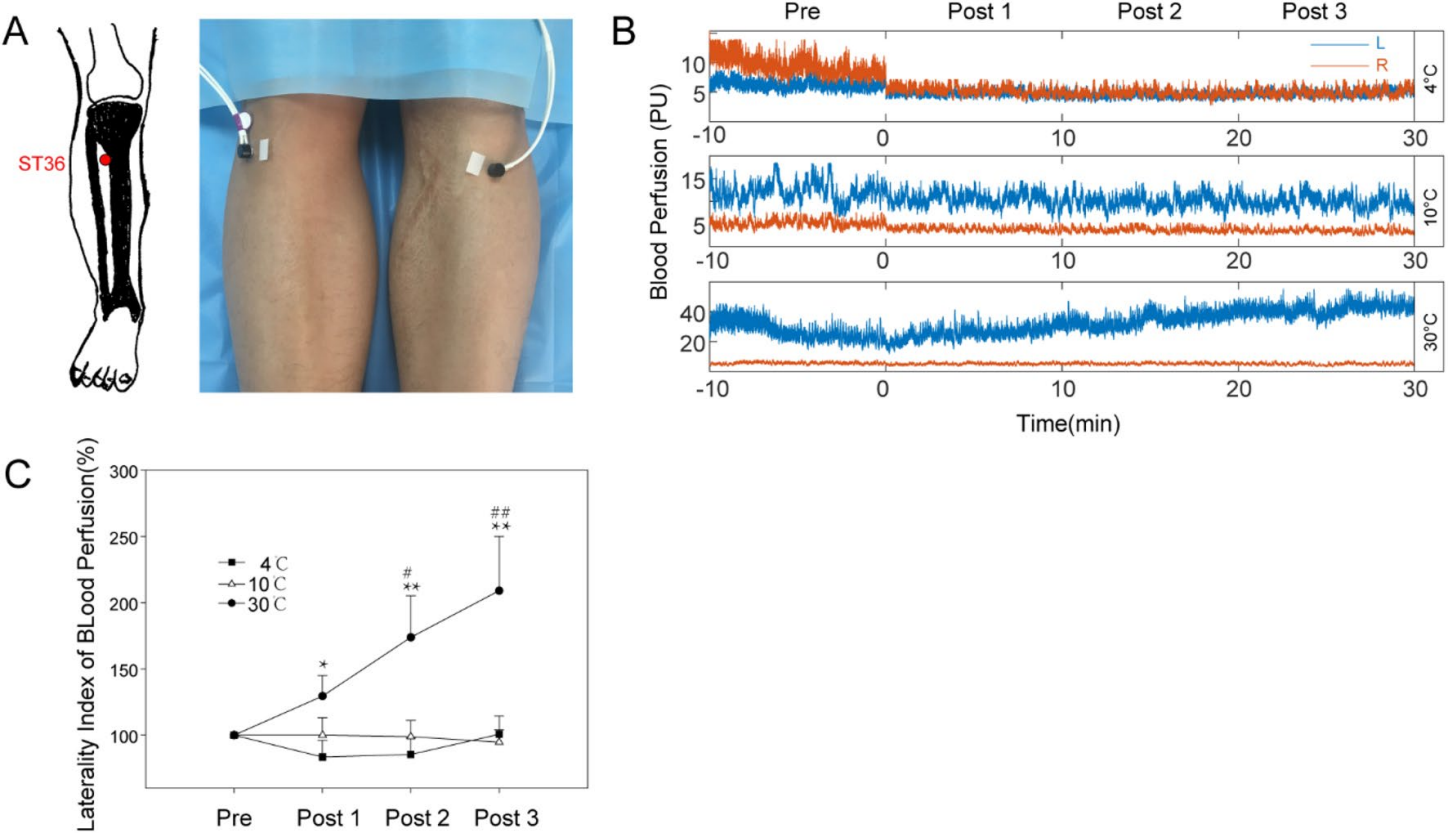
Blood perfusion on both sides. (**A**) Recording position at ST36. (**B**) Raw data of blood flux on both sides; (**C**) Laterality index of blood perfusion. *P_*corr*_ < 0.05, **P_*corr*_ < 0.01 (4 °C vs. 30 °C); #*P*_*corr*_ < 0.05; ##*P*_*corr*_ < 0.01 (10 °C vs. 30 °C);* P*_*corr*_: corrected *P* value. All values are reported as the mean ± standard error.

The average responses of blood perfusion to stimulation at different temperatures over time are shown in Fig. 4. The recording position of both sides for blood perfusion is shown in Fig. 4A, the raw data of bilateral blood perfusion are shown in Fig. 4B, and the laterality change in bilateral blood perfusion under different temperature stimulation conditions is shown in Fig. 4C. In the first 10 min after stimulation, there was a significant difference between the changes induced by 30 °C stimulation and 4 °C stimulation. At the second and third 10-min period after stimulation, there were significant differences in the changes induced by 30 °C stimulation compared tothose induced by 4 °C and 10 °C stimulation. However, there were no significant changes in the changes induced by 4 °C and 10 °C.

**Figure 5:**
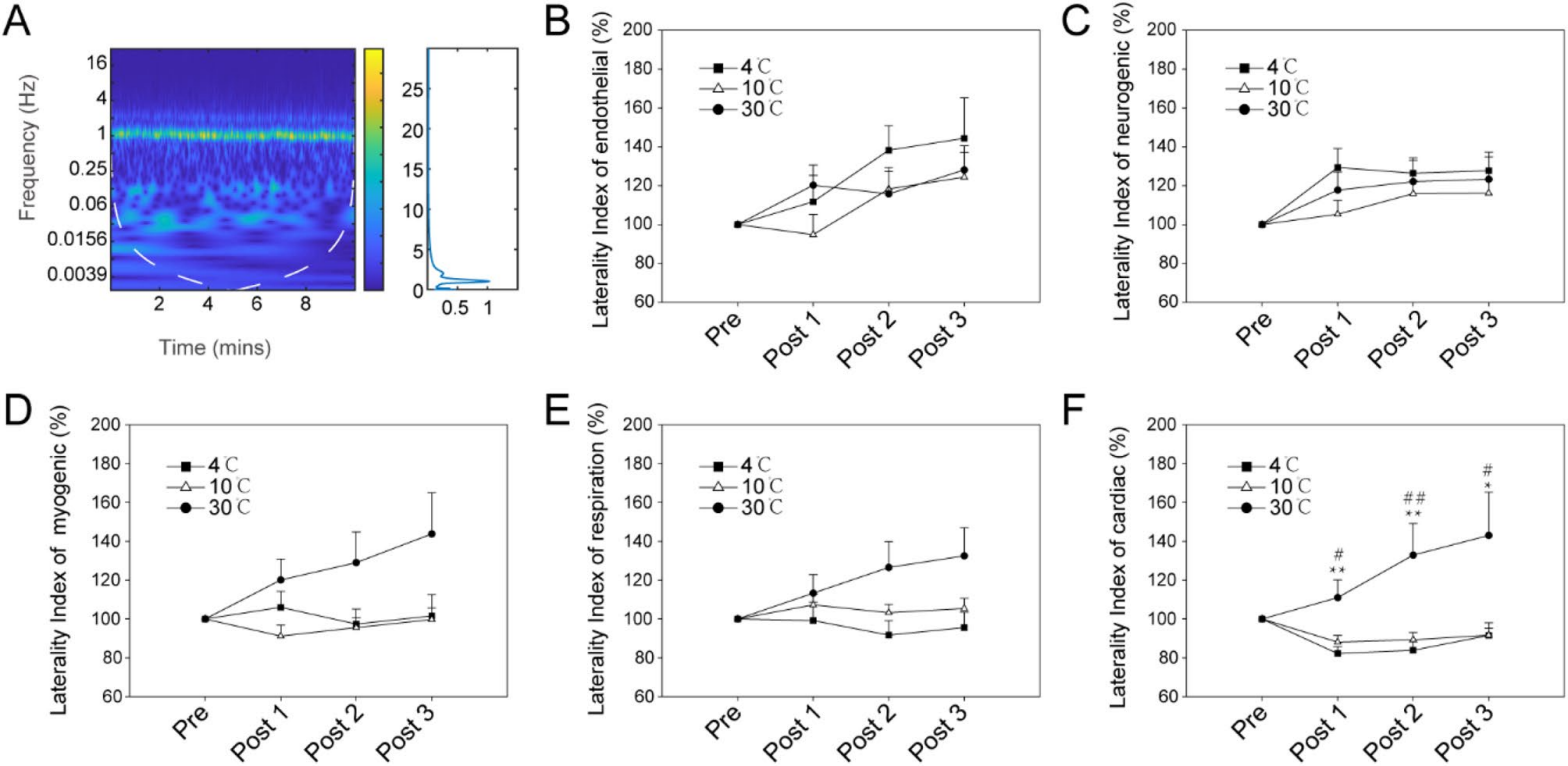
Laterality index for different components at different frequency intervals. (**A**) Wavelet analysis of blood flux; (**B**) Endothelial component (0.0095–0.02 Hz); (**C**) Neurogenic component (0.02–0.06 Hz); (**D**) Myogenic component (0.06–0.15 Hz); (**E**) Respiration component (0.15–0.4 Hz); (**F**) Cardiac component (0.4–1.6 Hz). Main effect of stimuli: *F*_(2,57)_ = 6.085, *P* = 0.00403; main effect of time: *F*_(3,171)_ = 3.159, *P* = 0.0261; interaction effect: *F*_(6,171)_ = 5.1, *P* < 0.001. Power > 0.95. Post hoc analysis reveals a significant increase for 30 °C from the first session. *P_*corr*_ < 0.05; **P_*corr*_ < 0.01 (4 °C vs. 30 °C); #*P*_*corr*_ < 0.05; ##*P*_*corr*_ < 0.01 (10 °C vs. 30 °C); *P*_*corr*_: corrected *P* value. All values are reported as the mean ± standard error.

Because the blood flow signal comprises many components, bilateral blood flow was transformed by wavelet (Fig. 5A), and the laterality index in different frequency intervals was calculated. The results showed that the difference was mainly reflected in frequency V (0.4–1.6 Hz) (Fig. 5F). There was no significant difference between other frequency intervals (Fig. 5B–E).

**Figure 6:**
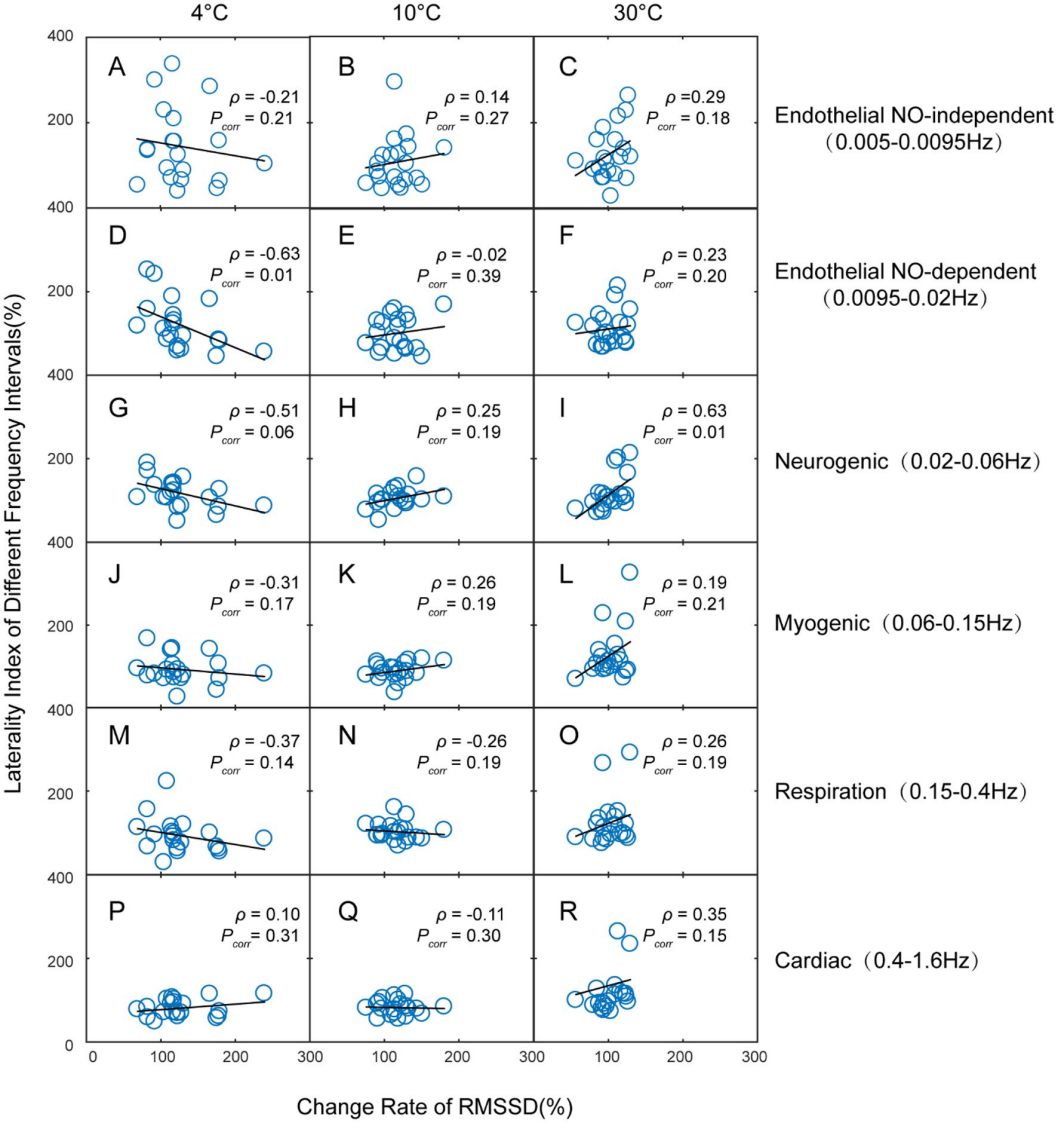
Relationship between laterality index of different frequency intervals and change rate of RMSSD. (**A**)–(**C**) Endothelial NO-independent; (**D**)–(**F**) Endothelial NO-dependent; (**G**)–(**I**) Neurogenic component; (**J**)–(**L**) Myogenic component; (**M**)–(**O**) Respiration component; (**P**)–(**R**) Cardiac component. ρ, Spearman’s correlation coefficient; *P*_*corr*_, corrected *P* value. RMSSD, root mean square of successive differences.

To further analyze the possibility of blood flow laterality, we analyzed the correlation between these parameters. First, we confirmed that there was no correlation between the DF and RMSSD (Figure S1) at different temperature stimulation. There was also no apparent correlation between the laterality index and the DF (Figure S2A-C) or RMSSD (Figure S2D-F). After wavelet transformation, we found that there was a negative correlation between RMSSD and the laterality index of endothelial NO-dependent component at 4 °C stimulation (Fig. 6D), which was independent of other components (Fig. 6A, G, J, M and P). There was a more positive correlation between RMSSD and the laterality index of neurogenic component (Fig. 6I) compared to other components (Fig. 6C, F, L, O and R) at 30 °C stimulation. There was no correlation between the laterality index of each of the components and RMSSD at 10 °C (Fig. 6B, E, H, K, N and Q). However, there was no correlation between the laterality index and DF in any components (Figure S3).

## Discussion

The main significance of current work is that there was response laterality of blood perfusion in the lower extremities after drinking saline at different temperatures, which means that autonomic nerve function is different between the left and right side of the body in the process of blood flow regulation. The present study demonstrated that drinking saline at different temperatures produced changes in cutaneous vascular movement, which means that regulation of cutaneous vascular might play an important role in water temperature digestion. The low-frequency component of blood flow mainly reflects local changes, while the high-frequency component mainly reflects systemic changes. In the current study, the skin blood flow regulation caused by gastrointestinal temperature stimulation was systemic, so the changes were mainly reflected in the high-frequency components.

A previous study^38^ indicated that the water effect does not depend on gastric distension, but on the water osmolality, which could cause an autonomic cardiovascular response in humans through osmotic sensing nerve fibers in the intestinal or portal circulation^5,38^. However, in the current study, osmotic pressure of saline at different temperatures was the same, and therefore it is difficult to conclude that the change in sympathetic tone was caused by osmolality.

In peripheral blood flow, small changes in cutaneous vascular blood perfusion can shift cardiovascular control. A decrease in the skin blood perfusion can lead to relative changes of sympathetic activation in peripheral blood flow^39^, which can be explained by the activation of temperature sensitive vagal afferent fibers found in the digestive tract^40^. In addition to spinal pathway afferent^41^, vagal afferent play an important role in the transmission of sensory information from the upper gastrointestinal tract to the central nervous system^42,43^. In Particular, in the proximal gut, the vagal afferents are more prevalent^18^. Although there are few studies on the response of vagal nerve to temperature sensors, some studies have shown that cold and warm stimulation of the cat esophagus, antrum and duodenum can induce a response from afferent neurons in the nodose ganglia, however, there is no response to chemical and mechanical stimulation^19,20^. This indicates that in the gastrointestinal tract, there is a special vagal afferent pathway that deals with temperature stimulation. Water intake seems to activate different gastrointestinal vagal afferent fibers in a temperature dependent manner and may affect subsequent cardiac vagal tone^6^. As shown in Fig. 6, there is no direct linear relationship between bilateral blood flow laterality and RMSSD, but after decomposing the blood flow components, it was found that there is a linear relationship between specific components and RMSSD. It is suggested that the regulation of peripheral blood flow induced by stimulation of different temperatures is selective. In the present study, the change trend of SkBF at bilateral ST 36 was different after ingestion of saline at different temperatures. As in previous findings^9,44^, this phenomenon is defined as laterality and can be described quantitatively by the laterality index.

Laterality tends to focus on the differentiation between left and right handedness and cerebral hemisphere function. However, little attention has been given to the lateralization of blood flow distribution. Our previous studies demonstrated that there are significant differences in the distribution and regulation of blood flow in the same parts of both sides of the body^9,10^, and such results have been confirmed by other research groups^45^. A previous study indicated that in young subjects, sympathetic vasoconstrictor activation after drinking water was not accompanied by an increase in arterial blood pressure^46^, which suggested that the change in vascular tone in the limbs may be partially compensated by opposing changes in other vascular beds^46^. Therefore, it can be inferred that the distribution of bilateral blood flow and its variation are asymmetric.

Furthermore, we believe that one of the adaptation mechanisms of the human body to changes in the external environment is the regulation of blood flow on the body’s surface as an instinctively dynamic process. When the human digestive tract is stimulated by external temperature, the body surface blood flow will respond to these stimuli. On the other hand, the body's response to external stimuli follows the principle of parsimony in order to achieve the lowest energy consumption. The asymmetry of bilateral blood flow regulation may reflect this principle.

The technology used in our current research is still relatively novel, so it was not possible to explore the possible mechanism impacting the findings of this study. In a future study, we will consider recording the EGG, EEG and SkBF at the same time. Then, the mechanism of skin blood flow regulation caused by gastrointestinal temperature stimulation can be discussed from the perspective of brain-gut-skin interaction. In this study, the laterality index did not reflect direction, thus the main limitation of this study was that the direct relationship between laterality and the measured variables not clear. Because the age of the recruited subjects was relatively homogenous, not all age groups were presented. Some reports suggest that there are gender differences in terms of both thermoregulation^47^ and blood flow regulation^48^. However, from the perspective of complexity analysis of blood flow signals, there is no gender difference in the blood flow between the left and right sides of the lower extremities under different body positions^24^. In the current study, the majority of the subjects recruited were female. Therefore, it was impossible to analyze the changes in the lower extremity blood flow laterality index in different age groups and different gender groups, which is another limitation of this study. In addition, the span of stimulation temperature conditions was too large. This was another limitation of this study.

## Conclusions

There was laterality of blood perfusion in the lower extremities after drinking saline at different temperatures.

## Supplementary Information

Supplementary Information.

## Data Availability

The datasets presented in this study can be found in online repositories. The names of the repository/repositories and accession number(s) can be found below: https://doi.org/10.6084/m9.figshare.14863581.v1. Subject’s characters and grouping information can be found in the file named subjects characters.xlsx. The blood flow data can be found in the file named “Blood_Data.rar”. The EGG data before intervention can be found in the file named “EGG_RawData_pre.rar”, and the post-intervention data can be found in the files named “EGG_RawData_post_1.rar” and “EGG_RawData_post_2.rar”. The EGG data can be imported into Matlab and then spectrum analysis can be carried out with main_NaCl_EGG.m. The blood perfusion data can be imported into Matlab via the Blood_import.m file.

## Abbreviations

SkBF: Skin blood flux
DF: Dominant frequency
EDF: European data format
RMSSD: Root mean square of successive differences
ST 36: Zusanli acupoint
ECG: Electrocardiogram
EGG: Electrogastrogram
HRV: Heart rate variability
TCM: Traditional Chinese Medicine
Fig: Figure
SD: Standard deviation
SE: Standard error
ANOVA: Analysis of variance
