# Supplementary material for: Laterality of blood perfusion in the lower extremities after drinking saline at different temperatures
Source: Sci Rep. 2023 Jan 28;13:1586. doi: 10.1038/s41598-023-28758-y (PMC9884233; doi:10.1038/s41598-023-28758-y)
Supplement: Supplementary file 1 — Supplementary Information. [file 41598_2023_28758_MOESM1_ESM.docx]

**Laterality of Blood Perfusion in the Lower Extremities after Drinking Saline at Different Temperatures**

Shuyong Jia^1*^, Qizhen Wang^2*^, Hongyan Li^1^, Xiaojing Song^1^, Shuyou Wang^1^, Weibo Zhang^1^, Guangjun Wang^1^

1. Institute of Acupuncture and Moxibustion, China Academy of Chinese Medical Sciences, Beijing, China
2. Institute of Basic Research in Clinical Medicine, China Academy of Chinese Medical Sciences, Beijing, China

* contributed equally

Shuyong Jia: [shuyong6666@163.com](mailto:shuyong6666@163.com)

Qizhen Wang:18600759031@163.com

Hongyan Li: lhylhyz90@163.com

Xiaojing Song: [xts2010@163.com](mailto:xts2010@163.com)

Shuyou Wang: wangsy15@126.com

Weibo Zhang: [zhangweibo@hotmail.com](mailto:zhangweibo@hotmail.com)

Guangjun Wang: tjuwgj@gmail.com

Corresponding Author: Guangjun Wang(tjuwgj@gmail.com)


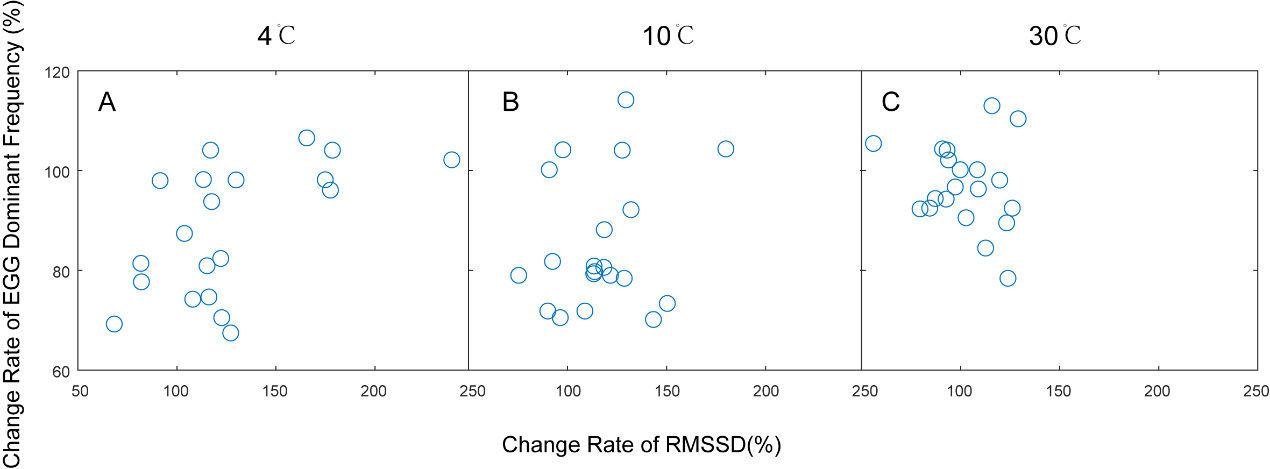


Figure S1. Relationship between EGG dominant frequency and ECG RMSSD at different temperature saline stimulation. (A) 4 °C stimulation. (B) 10 °C stimulation. (C) 30 °C stimulation.


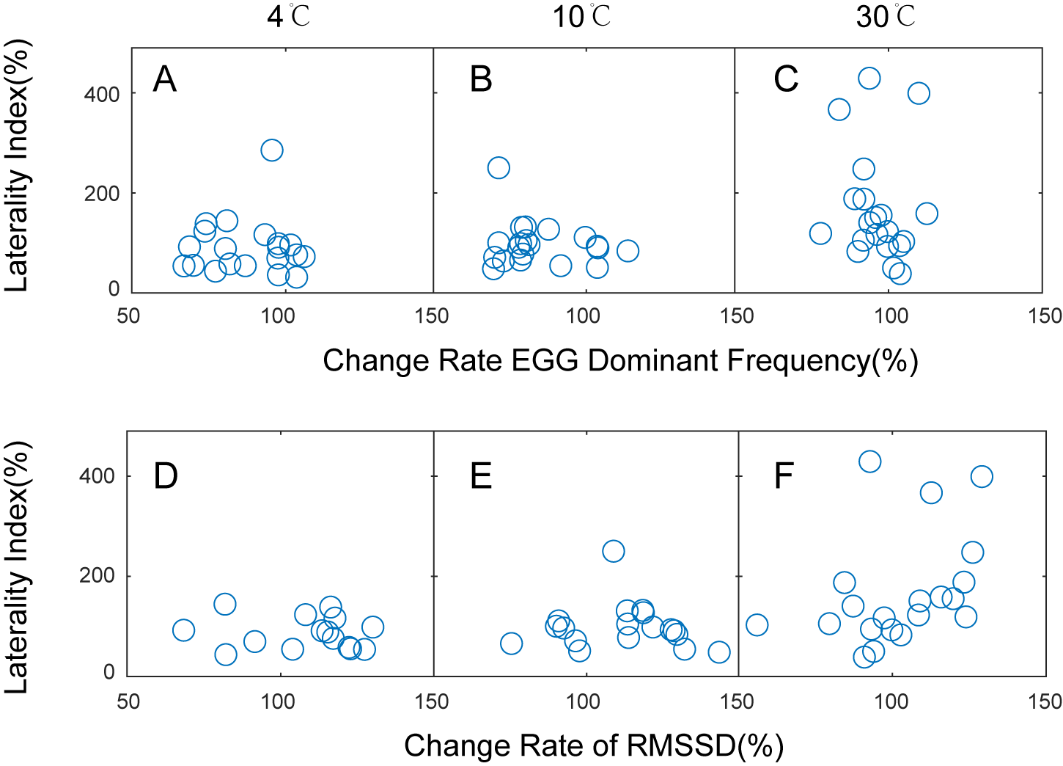


Figure S2. Relationship between laterality index and EGG dominant frequency or RMSSD. (A) LI vs DF at 4 °C. (B) LI vs DF at 10 °C. (C) LI vs DF at 30 °C. (D) LI vs RMSSD at 4 °C. (E) LI vs RMSSD at 10 °C. (F) LI vs RMSSD at 30 °C. DF, EGG dominant frequency; RMSSD, root mean square of successive differences; LI, laterality index.


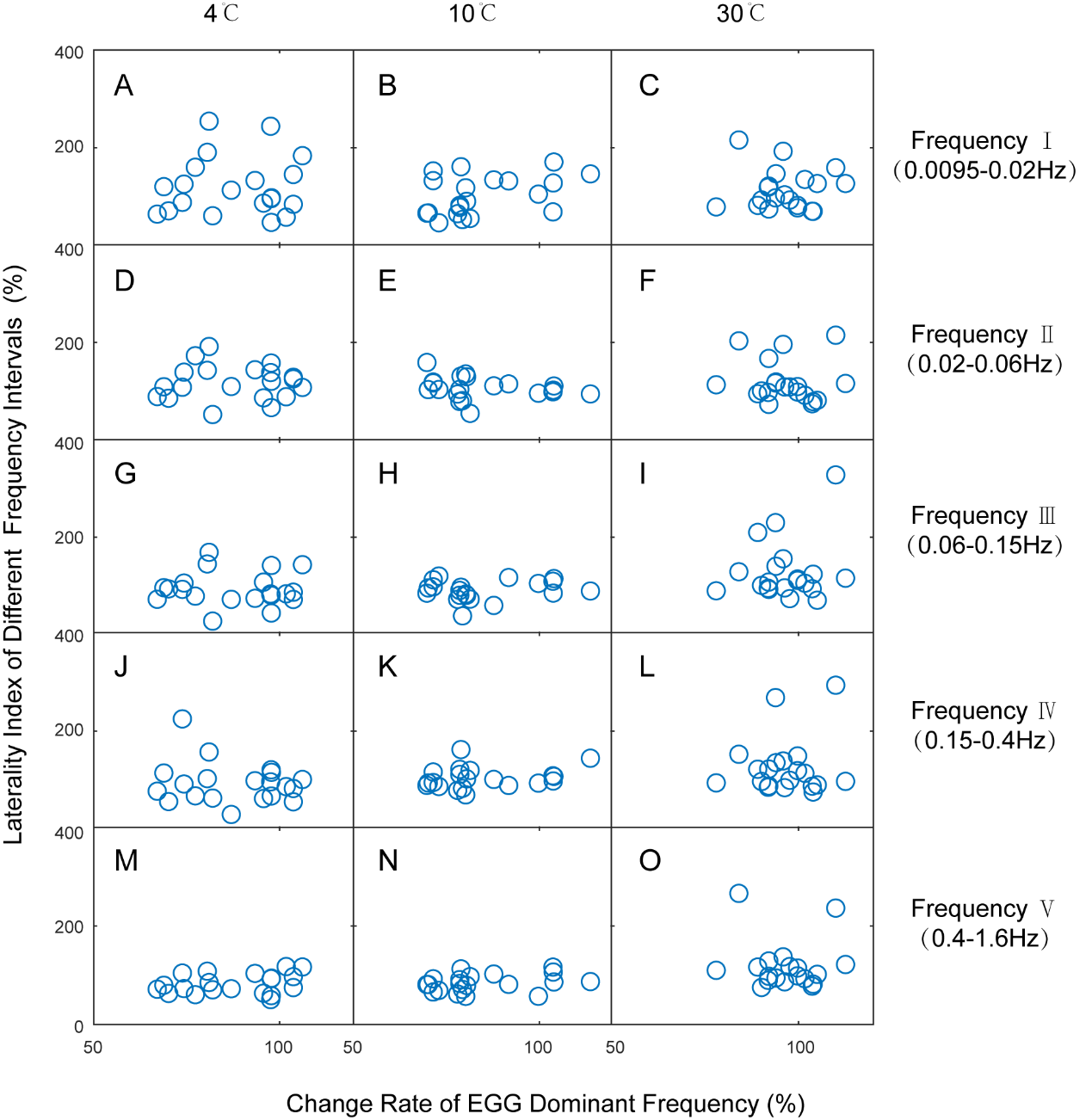


Figure S3. Relationship between laterality index of frequency intervals and EGG dominant frequency. (A). Frequency I at 4 °C. (B). Frequency I at 10 °C. (C). Frequency I at 30 °C. (D) Frequency II at 4 °C. (E) Frequency II at 10 °C. (F) Frequency II at 30 °C. (G) Frequency III at 4 °C. (H) Frequency III at 10 °C. (I) Frequency III at 30 °C. (J) Frequency IV at 4 °C. (K) Frequency IV at 10 °C. (L) Frequency IV at 30 °C. (M) Frequency V at 4 °C. (N) Frequency V at 10 °C. (O) Frequency V at 30 °C.
